# mHealth-assisted expiratory muscle strength training in Parkinson's disease patients: A proof-of-concept study

**DOI:** 10.1177/1877718X241296013

**Published:** 2025-01-14

**Authors:** Martin Srp, Álvaro Sánchez Ferro, Joaquim Ferreira, Ricardo Cacho, Laura Antunes, Raquel Bouça-Machado, Ota Gál, Martina Hoskovcová, Radim Kliment, Jan Mužík, Tiago A Mestre, Daniel Pérez Rangel, Evžen Růžička, iCARE-PD consortium

**Affiliations:** 1 Department of Neurology and Centre of Clinical Neuroscience, First Faculty of Medicine, Charles University and General University Hospital, Prague, Czech Republic; 2 HM CINAC, Hospital Universitario HM Puerta del Sur, Universidad CEU-San Pablo, Madrid, Spain; 3 Movement Disorders Unit, Neurology Department, Hospital Universitario 12 de Octubre, Madrid, Spain; 4 CNS – Campus Neurológico, Torres Vedras, Portugal; 5 Faculty of Biomedical Engineering, Czech Technical University, Prague, Czech Republic; 6 Parkinson's Disease and Movement Disorders Center, Division of Neurology, Department of Medicine, The Ottawa Hospital Research Institute, University of Ottawa Brain and Mind Institute, Ottawa, Canada

**Keywords:** Parkinson's disease, resistance training, respiratory muscles, mHealth, self-efficacy, patient compliance

## Abstract

**Background:**

Expiratory muscle strength training (EMST) is acknowledged for its therapeutic benefits in Parkinson's disease (PD), yet long-term adherence remains a challenge.

**Objective:**

The primary aim of this study was to assess the preliminary effects of EMST coupled with a mobile health app (SpiroGym) on self-efficacy and exercise adherence in PD patients. The secondary aim was to assess the usability of the SpiroGym app.

**Methods:**

This single-group, multicenter, multinational proof-of-concept study involved 63 PD patients across four tertiary PD centers. Participants were enrolled in either a 1-week (n = 35) or 24-week (n = 28) EMST program coupled with SpiroGym app. Self-efficacy was assessed using the Self-Efficacy for Home Exercise Program scale (SEHEPS) and exercise adherence was monitored by SpiroGym app. Usability was evaluated using the System Usability Scale.

**Results:**

Post-intervention, significant improvements in SEHEPS were observed in 1-week group (d = 0.48; p = 0.02) and 24-week group (d = 0.57; p = 0.002). Adherence rates in the 24-week PD patient group were high throughout the course of the study. Post-training SEHEPS was found to correlate (rho = 0.55; adjusted p = 0.016) with adherence to EMST during the non-supervised maintenance phase. The SpiroGym app exhibited high usability (>85th percentile score), with no significant differences noted between short-term and long-term use, indicating sustained user satisfaction.

**Conclusions:**

The results of our study suggest a promising role for SpiroGym app in supporting adherence to home-based EMST in PD patients. Nevertheless, future comparative studies are required to confirm SpiroGym's effectiveness.

## Plain language summary

People with Parkinson's disease (PD) can have trouble sticking with long-term exercise programs. This study looked at using a smartphone app called SpiroGym to see if it could help people with PD with their home breathing exercises. We included 63 patients with PD from four different treatment centers. Patients were divided into two groups: one used the SpiroGym app for 1 week, and the other for 24 weeks. We wanted to see if the app helped patients feel more confident about exercising at home and if breathing exercises were performed regularly. We also checked how easy the app was to use. After using the app, both groups reported feeling more confident about doing their home breathing exercises. The group that used the app for 24 weeks regularly did their exercises throughout the study. Participants rated the app highly for ease of use, regardless of how long they used it. In conclusion, the SpiroGym app might be a potentially helpful tool for people with PD to maintain their breathing exercises at home. However, future studies are needed to confirm these findings.

## Introduction

In the non-pharmacological management of Parkinson's disease (PD), expiratory muscle strength training (EMST) has emerged as a beneficial intervention, positively impacting key clinical aspects such as dysphagia,^1^ dystussia,^2^ hypokinetic dysarthria,^3^ and drooling.^4^ Given PD's progressive nature, long-term adherence to EMST programs is crucial to sustain its therapeutic benefits. However, individuals with neurological disorders frequently encounter challenges in adhering to prescribed exercise protocols, particularly in their home environments.^5^ As EMST is a home-based therapy, ensuring long-term adherence becomes a critical issue for many PD patients. This challenge is further intensified by the dysregulation of dopamine systems that occurs with the progression of PD, which can lead to decreased motivation, loss of interest in pleasurable activities, and a more negative perception of one's ability to exercise, thereby potentiating non-adherence to EMST.^6^ To date, no study has tracked long-term non-supervised EMST adherence in PD patients. However, a study by Sørensen (2018) in a non-neurological population showed that conventionally led, non-supervised respiratory training resulted in non-adherence to the training regimen.^7^ This finding aligns with our clinical experience regarding long-term non-supervised EMST adherence rates in PD patients. Among the factors associated with exercise behavior, self-efficacy stands out as the most consistent predictor of exercise adherence across diverse populations,^8–11^ including PD patients.^12^ Self-efficacy represents an individual's belief in the ability to successfully accomplish tasks that contribute to achieving the goals.^13^ Intervention programs aimed at increasing self-efficacy for exercise have revealed higher adherence rates and reduced dropout compared to control groups.^14,15^ A systematic review and meta-analysis have specifically emphasized that techniques involving feedback on past performances, or the performances of others, yield the highest levels of exercise self-efficacy.^16^ In today's high-tech era, mobile technology offers unique opportunities to provide tailored feedback and promote self-motivation.^12^ Building on these facts, we developed SpiroGym, a smartphone mobile health (mHealth) app, to monitor individual EMST performance, provide direct feedback on training performance, and track progress over time. In a prior study,^17^ we demonstrated the feasibility of EMST coupled with the SpiroGym app in PD patients. Moreover, recommendations from patients in a prior study^17^ guided us in co-designing and adapting the SpiroGym app to meet individual patient needs, representing a crucial milestone in the implementation of mHealth technologies.^18^ The subsequent step is conducting a proof-of-concept study that examines the preliminary impact of the SpiroGym app on patient self-efficacy and adherence.

Therefore, the primary aims of this multicentric study were to explore the impact of EMST coupled with the SpiroGym app on self-efficacy and to examine its correlation with adherence to a home-based EMST program. The secondary aim was to verify the usability of the modified SpiroGym app.

## Method

### Study design

A multicenter-multinational prospective proof-of-concept study was carried out across four tertiary PD centers: Charles University and General University Hospital, Czech Republic; Hospital Universitario HM Puerta del Sur, Spain; Ottawa Hospital Research Institute, Canada; and CNS—Campus Neurológico, Portugal. The study employed a dual-duration approach, consisting of two groups: 1) a 1-week group designed to preliminarily assess short-term changes in exercise self-efficacy and SpiroGym app usability; 2) a 24-week group aimed at (1) preliminary evaluation of exercise self-efficacy and its correlation with adherence to the EMST and (2) SpiroGym app usability. At the Charles University center in the Czech Republic, patients had the option to be enrolled in either a 1-week or a 24-week training duration, with the allocation to groups based on individual patient preferences. In other centers, recruitment was solely for the 1-week intervention duration. The study team considered the 1-week duration sufficient for participants to acclimate to the EMST routine and the SpiroGym app, allowing for an initial evaluation of the technology's user-friendliness and immediate impact on exercise self-efficacy. The 24-week duration was considered sufficient to explore the long- term adherence to EMST and its correlation with exercise self-efficacy.

### Participants

Patients were enrolled at the study centers based on the following criteria: 1) a diagnosis of PD using the UK Brain Bank criteria.^19^ The exclusion criteria were 1) suspected parkinsonism due to causes other than idiopathic PD, 2) a diagnosis of dementia or major psychiatric illness associated with psychosis (as recorded in the patient's case file), and 3) previous experience with EMST. The study was approved by the ethics committee of each participating center, and all participants provided written informed consent prior to participating in the study. All procedures were in accordance with the Declaration of Helsinki.

### Study outcome parameters

The exercise self-efficacy was assessed using the self-efficacy for home exercise program scale (SEHEPS). The SEHEPS is the only published scale specifically designed to assess self-efficacy for home exercise programs.^20^ A patient's self-efficacy score is the total sum of the 12 items, ranging from 0 to 72, where higher scores signify greater exercise self-efficacy. A SEHEPS score of less than 59 points identify individuals at risk of non-adherence (defined as <70% of exercise adherence) to home exercise programs.^20^ At baseline, patients completed the SEHEPS based on their confidence in undertaking any long-term home exercise program. Subsequently, at either week 1 or week 8, depending on group allocation, patients completed the SEHEPS again, focusing on their confidence in continuing long-term EMST with the SpiroGym app at home.

Adherence to the EMST program in 24-week group was assessed by comparing the recorded total number of forceful expirations, as documented by the SpiroGym app, against the prescribed number (1000 EMST maneuvers for the initial 8-week phase and 800 EMST maneuvers for the 16-week maintenance phase).

The system usability scale (SUS) is a standardized questionnaire widely used for evaluating user-perceived usability,^21^ including its application in assessing mHealth app.^22^ The questionnaire consists of 10 items, with responses ranging from “strongly agree” to “strongly disagree.” The overall score ranges from 0 to 100, with scores above 68 points considered as above average user-perceived usability. Utilizing the Sauro–Lewis curved grading scale, these scores are then converted to a normative percentile rank and assigned usability grades from A to F, where A represents excellent usability and F indicates poor usability.^23^

### Assessment visits

Participants in a 1-week group underwent two SEHEPS evaluations (baseline and post-1-week EMST) and completed the SUS evaluation for SpiroGym app after 1 week of EMST. Participants in a 24-week group underwent two SEHEPS evaluations (baseline and post-8-week EMST) and completed the SUS evaluation for SpiroGym app after 24 weeks of EMST. The test-retest reliability of SEHEPS was assessed in patients from the 24-week group. For this purpose, SEHEPS assessments were conducted in two separate testing sessions, with a one-week interval between them, before starting the EMST regimen. Patient adherence rates in the 24-week group were collected from the SpiroGym app at week 8 and week 24. A typical assessment visit lasted approximately 30 min. All examinations were carried out in the ON medication state.

### Treatment

#### SpiroGym app

The SpiroGym app evaluates real-time training data using a microphone attached to an expiratory muscle trainer. During a correct performance of the expiratory manoeuvre with the expiratory muscle trainer, the exhalation valve opens, and the air flow increases the sound level detected by the microphone. The SpiroGym app provides real-time visual feedback on the smartphone screen, displaying a curve representing the current sound level. Additionally, the SpiroGym app enables patients to review data from previous training sessions and creates a training diary. Further details about the SpiroGym app are provided in the study.^17^

#### Expiratory muscle strength training

**Figure 1.**
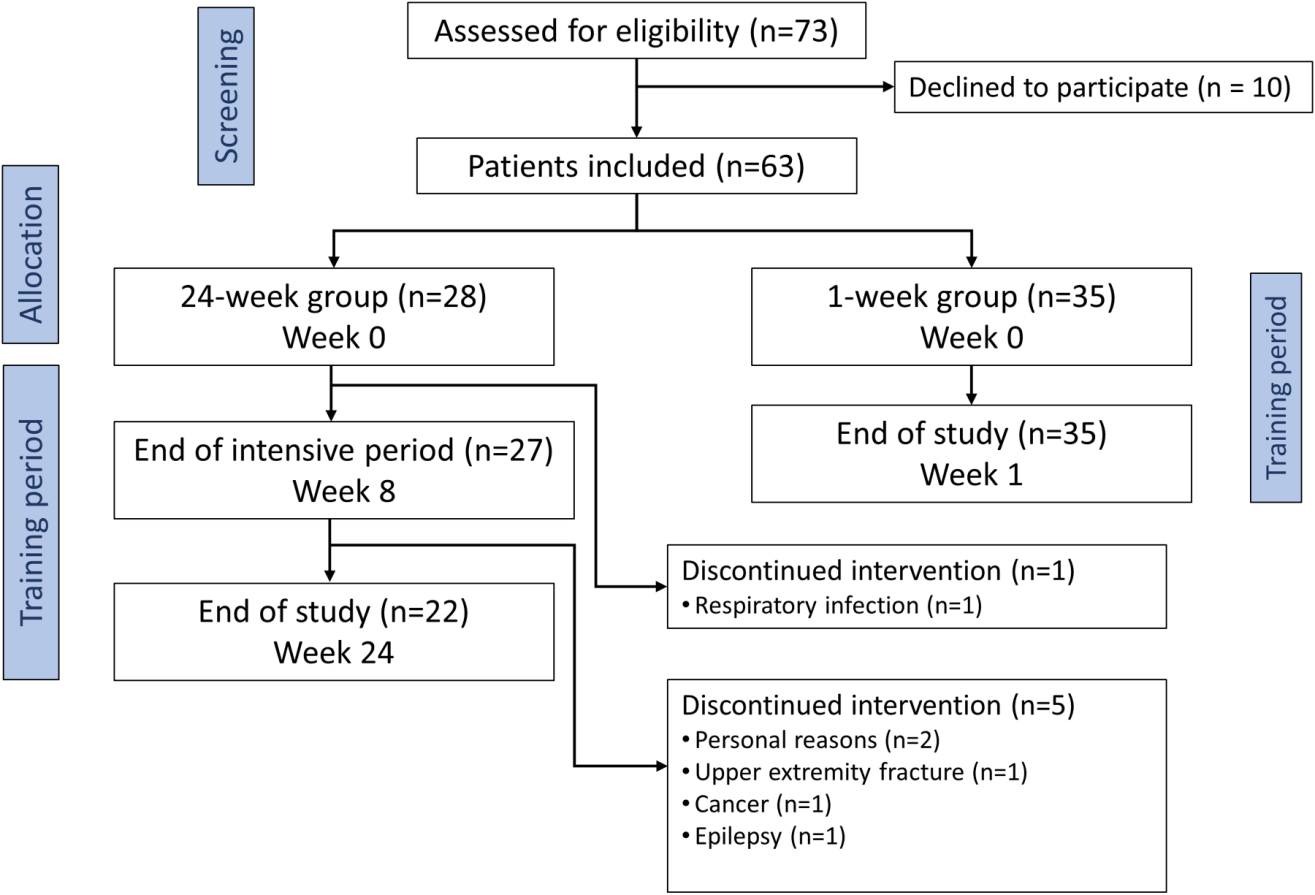
Study flowchart.

Patients performed EMST utilizing the expiratory muscle trainer (EMST150; Aspire Products, LLC, United States) for 1 week in their home environment. The EMST150 device was set up following the manufacturer's instructions (emst150.com). Participants were instructed to perform five sets of five forceful expirations five days a week, following the conventionally recommended frequency for EMST.^1,2^ Participants in the 24-week group were instructed to perform five sets of five forceful expirations five days a week for the initial 8 weeks. This phase included bi-weekly outpatient visits with a research physiotherapist for EMST150 adjustment (increasing trainer resistance), checking training performance and providing motivational support for adherence to the EMST protocol. Following the initial 8 weeks, patients continued with EMST coupled with the SpiroGym app for an additional 16 weeks without scheduled visits. During this period, participants were instructed to perform five sets of five forceful expirations twice a week, with the option to exercise more frequently if desired (for the study flowchart, see Figure 1). This design was based on the recommendation from.^24^

### Statistical analysis

To analyse differences in the observed variables between baseline and post-training, the non-parametric paired Wilcoxon test, and the McNemar test for categorical variables, were utilized. Differences between groups of patients were analysed using the non-parametric Mann-Whitney U-test. To evaluate correlations among the observed variables, the Spearman correlation coefficient was used. P-values were adjusted for multiple testing by the Holm method. To assess the test-retest reliability, an intraclass correlation coefficient (ICC) was used. Adjusted p-values less than 0.05 were considered statistically significant. Analyses were performed using the R statistical package, version 4.3.2.

## Results

A total of 63 PD patients participated in the study. A summary of demographic and clinical characteristics is provided in Table 1. Among the enrolled patients, 35 underwent a 1-week of EMST coupled with the SpiroGym app, while 28 participated in the 24-week regimen. In the 24-week group one patient discontinued the program within the initial 8-week phase, and an additional five discontinued during the 16-week maintenance period (detailed reasons are provided in Figure 1). In both the 1-week and 24-week groups, there were no technical issues with the SpiroGym app during EMST sessions or in post-training data acquisition.

**Table 1.** Demographic and clinical characteristics of the study sample.

	1-week group (n = 35)	24-week group (n = 28)
Age (range) in y	69 (61–72)	68 (58–76)
Gender		
*Female*	15	12
*Male*	20	16
Disease duration (y)	6 (4–10)	5.5 (3–17)
Caregiver status (n)		
*No caregiver*	30	26
*Informal*	5	2
*Formal*	0	0
Hoehn & Yahr scale (n)		
*Stage I.*	2	4
*Stage II.*	29	21
*Stage III.*	4	3
*Stage IV.*	0	0
MDS-UPDRS III	18 (14–31)	18.5 (9–31)
Muscle-strengthening activity (n) ^a^		
*Exercisers*		4
*Non-exercisers*		24

Values are median (interquartile range)

MDS-UPDRS III: Movement Disorder Society Unified Parkinson's Disease Rating Scale (Motor examination)

^a^ For the 24-week group, in addition to the overall demographic and clinical characteristics of the study sample, information regarding the regularity of muscle-strengthening activity in the past 6 months was collected. Based on the minimum activity recommendations,^36^ patients were categorized as either exercisers (engaging in muscle-strengthening activities ≥ 2 times per week with major muscle groups) or non-exercisers (< 2 times per week).

### SEHEPS

The test-retest reliability of SEHEPS was excellent [ICC = 0.96 (95% CI: 0.91–0.98); n = 28]. No correlations were found between the baseline SEHEPS scores and the demographic or clinical characteristics of the study participants (adjusted p values: 0.187–1.000). In the 1-week group, there was a significant increase [d = 0.48; 95% CI (0.18–0.89); p = 0.02] in SEHEPS score from baseline [median 48.0 points (IQR 34.5–54.5)] to post-testing [median 55.5 points (46.8–60.3)]. In the 24-week group, there was a significant increase [d = 0.57; 95% CI (0.25–0.92); p = 0.002] in SEHEPS score from baseline [median 43.5 points (IQR 33.0–55.3)] to post-testing [median 56.0 points (38.8–66.3)]. The difference in SEHEPS scores changes between the 1-week and 24-week groups was not statistically significant (p = 0.234).

### Adherence

Throughout the initial 8 weeks, patients from the 24-week group completed a median of 1000 (IQR 975–1000) out of 1000 prescribed expiratory maneuvers. Between weeks 8 to 24, the median number of completed expiratory maneuvers was 848 (IQR 781–1388) out of 800 prescribed. Eleven (50%) participants completed more than the 800 prescribed expiratory maneuvers. The SEHEPS score at 8 weeks was significantly correlated (rho = 0.55; adjusted p = 0.016) with the number of executed expiratory maneuvers performed in the non-supervised period from week 8 to week 24. No significant correlation was identified between the baseline SEHEPS score and the number of executed expiratory maneuvers during the semi-supervised intensive period (week 0 to week 8) and the non-supervised (week 8 to week 24) period (rho = 0.03; adjusted p = 0.883 and rho = 0.19; adjusted p = 0.781, respectively).

### The system usability scale

In the 1-week group, the median SUS score received an A + grade, at 85.0 points (IQR: 75.0–90.0), corresponding to the 96th–100th percentile range. The 24-week group attained a median SUS score of an A- grade, at 80.0 points (IQR: 65.0–85.0), falling within the 85th–89th percentile range. The difference in SUS score between the 1-week and 24-week group was not statistically significant (adjusted p = 0.185). No significant correlation was found between the SUS and the demographic and clinical characteristics of study participants (adjusted p values: 0.151–1.000).

## Discussion

This is the first study that tested the effect of an mHealth solution on exercise self-efficacy and adherence to EMST in PD patients. The results demonstrate that EMST, coupled with the SpiroGym app, significantly increased PD patients’ pretraining exercise self-efficacy for a home-based program. In patients who underwent the 24-week EMST program with SpiroGym app, adherence to prescribed exercises was exemplary. Moreover, post-training self-efficacy correlated with adherence during the non-supervised maintenance phase, highlighting the critical role of self-efficacy in sustaining long-term adherence to EMST. While the results are promising, they should be considered preliminary. Our study did not examine self-efficacy related to conventional EMST without SpiroGym app, so the effects solely attributable to the SpiroGym app cannot be conclusively distinguished. Therefore, these outcomes should be viewed as an initial step, confirming the promising effectiveness of SpiroGym app, while recognizing the need for further research.

Consistent with the literature positing moderate effects (d = 0.43) of feedback about past performance on exercise self-efficacy,^16^ SpiroGym's real-time feedback mechanism aligns our results (d = 0.48–0.57) with these findings. Interestingly, our study found no significant differences in exercise self-efficacy changes between different training durations and regimens (a semi-supervised 8-week and a non-supervised 1-week EMST). This aligns with the findings that the effect of interventions on exercise self-efficacy tends to diminish once participants learn to exercise properly and develop sufficient experience.^25^ In the case of our study, it appears that one week was sufficient for participants to acquire effective exercise habits, which may explain the absence of a progressive increase in exercise self-efficacy perception in 24-week group. This indicates that therapist-led training may not be essential for enhancing patients’ exercise self-efficacy in EMST with the SpiroGym app. This observation aligns with our future intention of having the SpiroGym app-led EMST program run independently, without regular therapist visits. This approach aligns with recent reviews,^12^ which suggest that future research in PD should investigate whether an mHealth app can effectively substitute traditional trainer-led interventions. Inspired by the success of home-based pulmonary rehabilitation programs delivered via mHealth technology, which have proven as effective as center-based programs,^26^ and the demonstrated feasibility of home-based EMST delivery, including re-adjustments of training load, via telehealth in movement disorders patients,^27^ we see a strong precedent for the effectiveness of SpiroGym-led EMST regimen for PD patients.

The preliminary findings on adherence in our 24-week study group are promising, showing that even patients not regularly engaged in strength training (83% of participants), managed to maintain excellent adherence to the 24-week EMST regimen. The dropout rate of 21% in the 24-week group was attributed to medical conditions and personal circumstances unrelated to the EMST intervention. Most EMST studies have not reported results on PD patient's adherence,^1,4,28–30^ making it difficult to compare our adherence results with other studies. Only one study has fully reported on EMST compliance among PD patients,^2^ where patients completed 97% of prescribed exercise over 5 weeks, which is similar to our 8-week adherence results. However, 22% of the included PD patients in the Troche 2022 study did not return exercise logs, raising questions about their adherence rates.^2^ To our knowledge, the longest monitored EMST protocol to date involved a single PD patient over a 20-week period,^31^ but it lacked adherence data information.

We did not find a correlation between baseline SEHEPS scores and adherence during either the intensive or maintenance periods. However, a significant correlation was found between exercise self-efficacy measured at the 8-week visit and exercise adherence in the 16-week phase. This divergence aligns with Bandura's assertions, where mastery experience, as one of the main sources of self-efficacy, can rapidly recalibrate pretraining beliefs (as observed in the 1-week group).^13^ This supports the notion that self-efficacy beliefs, developed during the intervention in response to exercise experiences, predict the maintenance of the exercise regimen more reliably than baseline beliefs. This is in line with the results of other studies.^8,32^

So far, all EMST studies have tracked patient exercise adherence using subjective methods, such as self-reporting in exercise diaries. However, this approach has its challenges.^33^ A study^34^ highlighted considerable variability in the accuracy of PD patients’ self-reported diaries, with a tendency to report prescribed exercises rather than actual ones completed. In contrast, the SpiroGym app offer a more objective method of measurement by processing and logging training data from previous sessions, ensuring that these records cannot be altered by users. Given that the SpiroGym app recorded adherence without any technical problems throughout the entire 24-week training period in all patients, its precise tracking is particularly valuable for future research about exercise adherence.

The results of this study showed very good usability of the SpiroGym app among PD patients. No significant correlation was found between the SUS and the demographic and clinical characteristics of participants, suggesting good SpiroGym app usability across various levels of PD patients’ disease severity and age. Furthermore, the high usability, within the 85th–89th percentile range, after 24-week SpiroGym app usage, highlights the app's ability to maintain user engagement and satisfaction. This reinforces its value in long-term therapeutic regimens for managing PD.

The interpretation of this study's results should be considered within the context of its limitations. First, this study did not examine the effects of EMST without the SpiroGym app on exercise self-efficacy and adherence, preventing the distinction of the treatment effect of the SpiroGym app from the effect of EMST alone. A future fully powered randomized controlled trial is required, comparing a SpiroGym app with standard treatment. Second, the allocation to the 24-week study group was based on patient preferences, potentially skewing the adherence results due to varying motivation levels among patients. Third, although the 1-week basic study protocol was implemented at all centers, the 24-week option was investigated at only one center. This limits the understanding of the long-term potential of EMST with the SpiroGym app in diverse settings. Finally, although the usability results showed no association with disease severity, it must be acknowledged that the majority of patients enrolled had only mild to moderate PD. The cohorts in previous EMST studies were similarly skewed toward milder stages of PD.^2,28,29^ Given that EMST is actually effective in dystussia, dysphagia and drooling that become more prevalent in the advanced stages of PD, further studies involving patients with advanced PD, who may have different problems and needs when using this device, will be needed. Future comparative studies should also consider assessing features of the sample, such as education, cognitive capacity, and depression/apathy. While our study focused on the impact of the SpiroGym app on self-efficacy and adherence, future research should test the hypothesis at hand that feedback through the app should enhance EMST and amplify its effects on clinical outcomes. In respiratory training, evidence already exists to support this.^35^ This approach could also prove beneficial for other types of respiratory training, such as inspiratory muscle strength training.

## Conclusions

The preliminary findings from this proof-of-concept study highlight the promising role of the SpiroGym mHealth technology in supporting home-based EMST for PD patients. Future studies with a comparative design are necessary to validate the efficacy of the SpiroGym in enhancing exercise self-efficacy and adherence to EMST.

## References

[1] Claus I Muhle P Czechowski J , et al. Expiratory muscle strength training for therapy of pharyngeal dysphagia in Parkinson's disease. Mov Disord 2021; 36: 1815–1824. 33650729 10.1002/mds.28552

[2] Troche MS Curtis JA Sevitz JS , et al. Rehabilitating cough dysfunction in Parkinson's disease: a randomized controlled trial. Mov Disord 2023; 38: 201–211. 36345090 10.1002/mds.29268

[3] Tong T Ng M Yan N . Impact of expiratory muscle strength training (EMST) on phonatory performance in Parkinson’s patients [abstract]. Mov Disord 2016; 31: 288.

[4] Cocks N Rafols J Embley E , et al. Expiratory muscle strength training for drooling in adults with Parkinson’s disease. Dysphagia 2022; 37: 1525–1531. 35171321 10.1007/s00455-022-10408-6 PMC9643176

[5] Isernia S Pagliari C Jonsdottir J , et al. Efficiency and patient-reported outcome measures from clinic to home: the human empowerment aging and disability program for digital-health rehabilitation. Front Neurol 2019; 10: 1206. 31824398 10.3389/fneur.2019.01206 PMC6882300

[6] Stevens A Stanton R Rebar AL . Helping people with Parkinson disease build exercise self-efficacy. Phys Ther 2020; 100: 205–208. 31665447 10.1093/ptj/pzz160

[7] Sørensen D Svenningsen H . Adherence to home-based inspiratory muscle training in individuals with chronic obstructive pulmonary disease. Appl Nurs Res 2018; 43: 75–79. 30220368 10.1016/j.apnr.2018.07.005

[8] Brassington GS Atienza AA Perczek RE , et al. Intervention-related cognitive versus social mediators of exercise adherence in the elderly. Am J Prev Med 2002; 23: 80–86. 12133741 10.1016/s0749-3797(02)00477-4

[9] Jancey J Lee A Howat P , et al. Reducing attrition in physical activity programs for older adults. J Aging Phys Act 2007; 15: 152–165. 17556782 10.1123/japa.15.2.152

[10] Oman RF King AC . Predicting the adoption and maintenance of exercise participation using self-efficacy and previous exercise participation rates. Am J Health Promot 1998; 12: 154–161. 10176088 10.4278/0890-1171-12.3.154

[11] Rhodes RE Martin AD Taunton JE . Temporal relationships of self-efficacy and social support as predictors of adherence in a 6-month strength-training program for older women. Percept Mot Skills 2001; 93: 693–703. 11806588 10.2466/pms.2001.93.3.693

[12] Schootemeijer S Van Der Kolk NM Ellis T , et al. Barriers and motivators to engage in exercise for persons with Parkinson’s disease. J Parkinsons Dis 2020; 10: 1293–1299. 32925106 10.3233/JPD-202247 PMC7739964

[13] Bandura A . Self-efficacy: toward a unifying theory of behavioral change. Psychol Rev 1977; 84: 191. 847061 10.1037//0033-295x.84.2.191

[14] Annesi J . Effects of a cognitive behavioral treatment package on exercise attendance and drop out in fitness centers. Eur J Sport Sci 2003; 3: 1–12.

[15] Burgess E Hassmén P Welvaert M , et al. Behavioural treatment strategies improve adherence to lifestyle intervention programmes in adults with obesity: a systematic review and meta-analysis. Clin Obes 2017; 7: 105–114. 28199047 10.1111/cob.12180

[16] Ashford S Edmunds J French DP . What is the best way to change self-efficacy to promote lifestyle and recreational physical activity? A systematic review with meta-analysis. Br J Health Psychol 2010; 15: 265–288. 19586583 10.1348/135910709X461752

[17] Srp M Korteová R Kliment R , et al. Expiratory muscle strength training in patients with Parkinson's disease: a pilot study of mobile monitoring application. Mov Disord Clin Pract 2021; 8: 1148–1149. 34631956 10.1002/mdc3.13313 PMC8485587

[18] Espay AJ Hausdorff JM Sánchez-Ferro Á , et al. A roadmap for implementation of patient-centered digital outcome measures in Parkinson's disease obtained using mobile health technologies. Mov Disord 2019; 34: 657–663. 30901495 10.1002/mds.27671 PMC6520192

[19] Hughes AJ Daniel SE Kilford L , et al. Accuracy of clinical diagnosis of idiopathic Parkinson's disease: a clinico-pathological study of 100 cases. J Neurol Neurosurg Psychiatry 1992; 55: 181–184. 1564476 10.1136/jnnp.55.3.181 PMC1014720

[20] Picha KJ Lester M Heebner NR , et al. The self-efficacy for home exercise programs scale: development and psychometric properties. J Orthop Sports Phys Ther 2019; 49: 647–655. 31291552 10.2519/jospt.2019.8779

[21] Lewis JR . The system usability scale: past, present, and future. Int J Hum Comput Interact 2018; 34: 577–590.

[22] Zapata BC Fernández-Alemán JL Idri A , et al. Empirical studies on usability of mHealth apps: a systematic literature review. J Med Syst 2015; 39: 1–19. 25600193 10.1007/s10916-014-0182-2

[23] Sauro J Lewis JR . Quantifying the user experience: Practical statistics for user research. Burlington, MA: Morgan Kaufmann, 2016.

[24] Sapienza C Hoffman B . Respiratory muscle strength training. San Diego: Plural Publishing, 2021.

[25] Higgins TJ Middleton KR Winner L , et al. Physical activity interventions differentially affect exercise task and barrier self-efficacy: a meta-analysis. Health Psychol 2014; 33: 891. 23957904 10.1037/a0033864 PMC4148031

[26] Chung C Lee JW Lee SW , et al. Clinical efficacy of mobile app–based, self-directed pulmonary rehabilitation for patients with chronic obstructive pulmonary disease: systematic review and meta-analysis. JMIR Mhealth Uhealth 2024; 12: e41753. 10.2196/41753 PMC10786334 38179689

[27] Sevitz JS Borders JC Dakin AE , et al. Rehabilitation of airway protection in individuals with movement disorders: a telehealth feasibility study. Am J Speech Lang Pathol 2022; 31: 2741–2758. 36279509 10.1044/2022_AJSLP-22-00063 PMC9911128

[28] Reyes A Castillo A Castillo J . Effects of expiratory muscle training and air stacking on peak cough flow in individuals with Parkinson’s disease. Lung 2020; 198: 207–211. 31720808 10.1007/s00408-019-00291-8

[29] Reyes A Castillo A Castillo J , et al. The effects of respiratory muscle training on peak cough flow in patients with Parkinson’s disease: a randomized controlled study. Clin Rehabil 2018; 32: 1317–1327. 29756459 10.1177/0269215518774832

[30] Troche M Okun M Rosenbek J , et al. Aspiration and swallowing in Parkinson disease and rehabilitation with EMST: a randomized trial. Neurology 2010; 75: 1912–1919. 21098406 10.1212/WNL.0b013e3181fef115 PMC2995389

[31] Saleem AF Sapienza CM Okun MS . Respiratory muscle strength training: treatment and response duration in a patient with early idiopathic Parkinson's disease. Neurorehabilitation 2005; 20: 323–333. 16403998

[32] Neupert SD Lachman ME Whitbourne SB . Exercise self-efficacy and control beliefs: effects on exercise behavior after an exercise intervention for older adults. J Aging Phys Act 2009; 17: 1–16. 19299835 10.1123/japa.17.1.1 PMC3740728

[33] Nagpal TS Mottola MF Barakat R , et al. Adherence is a key factor for interpreting the results of exercise interventions. Physiotherapy 2021; 113: 8–11. 34555674 10.1016/j.physio.2021.05.010

[34] Schmidt M Paul SS Canning CG , et al. The accuracy of self-report logbooks of adherence to prescribed home-based exercise in Parkinson’s disease. Disabil Rehabil 2022; 44: 1260–1267. 32762573 10.1080/09638288.2020.1800106

[35] Ribeiro R Brandão D Noronha J , et al. Breath-stacking and incentive spirometry in Parkinson’s disease: randomized crossover clinical trial. Respir Physiol Neurobiol 2018; 255: 11–16. 29727719 10.1016/j.resp.2018.04.011

[36] Nelson ME Rejeski WJ Blair SN , et al. Physical activity and public health in older adults: recommendation from the American College of Sports Medicine and the American Heart Association. Circulation 2007; 116: 1094. 17671236 10.1161/CIRCULATIONAHA.107.185650

